# MCred: multi-modal message credibility for fake news detection using BERT and CNN

**DOI:** 10.1007/s12652-022-04338-2

**Published:** 2022-07-27

**Authors:** Pawan Kumar Verma, Prateek Agrawal, Vishu Madaan, Radu Prodan

**Affiliations:** 1 Present Address: Lovely Professional University, Phagwara, India; 2 University of Klagenfurt, Klagenfurt, Austria; 3 MIT Art, Design and Technology University, Pune, India

**Keywords:** Fake news classification, Natural language processing, Deep learning, Dense network, Text classification, Convolutional neural network, Social media disinformation, Global semantic, local semantic

## Abstract

Online social media enables low cost, easy access, rapid propagation, and easy communication of information, including spreading low-quality fake news. Fake news has become a huge threat to every sector in society, and resulting in decrements in the trust quotient for media and leading the audience into bewilderment. In this paper, we proposed a new framework called **M**essage **Cred**ibility (MCred) for fake news detection that utilizes the benefits of local and global text semantics. This framework is the fusion of Bidirectional Encoder Representations from Transformers (BERT) using the relationship between words in sentences for global text semantics, and Convolutional Neural Networks (CNN) using N-gram features for local text semantics. We demonstrate through experimental results a popular Kaggle dataset that MCred improves the accuracy over a state-of-the-art model by 1.10% thanks to its combination of local and global text semantics.

## Introduction

News is any information to make the public aware of the events happening around them and which can affect them personally or socially. In recent years, online social media has become a common platform for news broadcasting for business, political, and entertainment purposes. Individuals use social media to search and consume news because of its ease, comfort, and fast propagation (Zhang and Ghorbani 2020). This commodity brought both constructive and destructive impacts. People tamper and scatter genuine information for their entertainment and benefits in the form of fake news (Bondielli and Marcelloni 2019). Fake news played a pivotal role in the 2016 US presidential election campaign following a large amount of false information spread on Facebook during its last three months (Allcott and Gentzkow 2017). This incident brought fake news to the attention of many industrial and research institutions for understanding and reducing this phenomenon.

*Fake news and social impact* Several researchers used the terms like false news, fake news, rumor, and disinformation interchangeably (Ajao et al. 2018; Bondielli and Marcelloni 2019; Lazer et al. 2018). There is no single universal definition of fake news (Zhou et al. 2019), however, we may define the term as any fabricated and deceitful news content that influences its readers to believe in something false. Klein and Wueller (2017) characterized fake news as the online distribution of false information purposefully or intentionally. While printed media was the only medium to spread fake news until a decade ago, online media became today the easiest way to spread low-quality news (Thota et al. 2018). Fake news can lead to a negative effect on politics, the economy, and public opinions. One popular fake news example was Barack Obama being harmed in a blast, which siphoned 130 billion dollars in stock (Rapoza 2020). Numerous fake news on COVID-19 pandemic over social networking sites (Kouzy et al. 2020) caused fear and misconception among the people. Recently, Tim Berners-Lee (Swartz 2020) stated that fake news has become the most upsetting thing over the Internet.

*News classification* Several researchers treated fake news as a binary classification (i.e. real or fake) (Shu et al. 2017; Sharma and Sharma 2021; Garg and Sharma 2020a), while others considered it as a multi-class classification (Karimi et al. 2018), regression, or clustering (Oshikawa et al. 2020) problem. An automated tool assists users in detecting and categorizing fake news according to three criteria, identified in the related work:

1. *Propagation-based* (Liu and Wu 2018; Liu et al. 2018) methods trace the spreading pattern of any news using people’s replies and share.
2. *User profile-based* (Shu et al. 2017) methods track the individuals’ behavior using their published, forwarded, or commented news including further analysis information like location, sexual orientation, followers, or friends.
3. *News content-based* (Zhou et al. 2020; Garg and Sharma 2020b; Zhang et al. 2020; Wang et al. 2021) methods are of two kinds:
  - *Syntactic-based* methods use linguistic and writing patterns like a number of special characters, nouns, or verbs to classify the news.
  - *Semantic-based* methods perform high-level representation and structure of the text in a given document.

### Method

We propose in this paper a novel *message credibility (MCred) multi-modal method* that approaches fake news as a binary classification problem. The method combines global text semantics relationship between words using bidirectional encoder representations from transformers (BERT) with local text semantics using *n*-grams features of a convolutional neural network (CNN) model. The MCred model uses global and local word embedding as a cue for the news classification validated using four datasets for training and testing purposes. We generated the CNN output by combining multiple *n*-gram features (i.e. a kernel size of two, three, and four). Finally, we combined the CNN and BERT outputs into a dense network to enhance the performance of MCred model. We achieved a 1.48% improvement in accuracy compared to related state-of-the-art methods.

### Outline

 The paper has six sections. Section 2 highlights the literature study. Section 3 explains the background of both machine learning (ML) and deep learning (DL). Section 4 describes the proposed MCred model comprising a BERT processing layer, A CNN processing layer, and a Dense net processing layer. Section 5 provides implementation details, followed by the evaluation results. Section 6 concludes the paper and highlights future work opportunities.

## Related work

Many researchers did survey on fake news detection and identified the prominent attributes, liable for the fake news classification (Sharma and Sharma 2019). We review in this section the state-of-the-art works on fake news detection into two categories: pattern-based and content-based. We conclude the section with a review of the research available for supporting fake news detection research.

### Pattern-based detection

Several researchers referred user profile-based features for fake news detection. Shu et al. (2017) investigated data mining and the correlation between the user profile features and the news genuineness and concluded with open fake news detection challenges. Singh et al. (2020) used attention-based LSTM to classify rumor and non-rumor tweets with thirteen linguistic and user profile features and achieved an F1-score of 88%. Horne and Adali (2017) developed support vector machine (SVM) model for fake news detection using three linguistics features categories: writing pattern, text complexity, and psychological. Similarly, Pérez-Rosas et al. (2018) manually built linguistic features from news and a trained an SVM model. Other researchers used reinforcement learning (Zhou et al. 2020) and fact checking (Vo and Lee 2019) for the news classification. Mangal and Sharma (2020) used the cosine similarity index approach for the reliable news prediction. They executed proposed model on 1000 news articles and achieved the accuracy of 91.07% with the assumption of 0.62 threshold. Sharma et al. (2019) concluded that generalizing linguistic features for fake news detection is a hard problem across different themes and domains. Singh et al. (2017) used a popular linguistic features package “Linguistic Analysis and Word Count (LIWC)”, and implemented a Z-score normalization technique with 80–20% training and testing set ratios. They compared several ML models and achieved the best results with 87% accuracy using SVM.

### Content-based detection

Safaya et al. (2020) proposed a BERT-CNN based model and compared F1-score value with five other state-of-the-art models. They concluded that BERT-CNN combination gives the improved result among all other models. They trained and tested their model on Arabic, Greek and Turkish tweets and claimed that their model might give improved result on other natural languages too. He et al. (2019) proposed a single-layer CNN model with BERT and evaluated on the Airline Travel Information Systems (ATIS) dataset. They achieved the accuracy of 98.54% but they explained that this model is suitable for short sentences only and the robustness of model can be improved after making some enhancements. Jwa et al. (2019) proposed exBAKE model for the articles classification in four categories i.e. agrees, disagrees, discusses and unrelated. For this, they used Daily Mail news as extra data for improved CNN training. They achieved the F1 score of 74.60% only. Guo et al. (2018) proposed a hierarchical bi-directional long short term memory (BLSTM) model and used an attention mechanism for rumor detection with a 93.4% accuracy on the Weibo dataset and 83.40% accuracy on the Twitter dataset. Ahn and Jeong (2019) used a fined tuned BERT for detecting fake news on a Korean dataset and achieved the area under the receiver operating characteristic curve score of 83.8%. Ahmed et al. (2017) implemented a term frequency and inverse document frequency (TF-IDF) extraction technique on six ML models. The linear SVM uni-gram with 50 thousand features achieved an experimental accuracy of 92%, while the linear regression uni-gram with the same number of features an 89% accuracy. O‘Brien et al. (2018) utilized a black-box DL framework for fake news detection and achieved an accuracy of 93.50%, which justified that CNNs work well for these types of problems. Singh and Sharma (2021) proposed a deep learning based multi-modal approach for the social media news classification. They used CNN for the image processing and RoBERTa for text processing; with this combination they achieved an accuracy of 85.3% on MediaEval (Twitter) dataset and 81.2% on Weibo dataset. Sharma et al. (2021) explained various existing tools and ways for fake news detection and also explained the role of fact checking websites in this classification task. They also executed LSTM and BiLSTM classifier on Kaggle dataset and concluded that with an accuracy of 91.51%, Bi-LSTM performed better than LSTM. *ii)* CNN processing layer requires large data to train and it is slower because of maxpool operation. Similarly at the testing phase, we require properly preprocessed and larger data. Khan and Alhazmi (2020) also used an ensemble technique to compare the performance of several ML models and achieved the highest accuracy of 90.70% using an AdaBoost random forest. Mersinias et al. (2020) proposed a novel class label frequency distance vectorization approach for fake news detection and found that logistic regression gives the highest accuracy of 97.52%. Kaliyar et al. (2020) used the GloVe word embedding model and deep CNNs for fake news detection and achieved an accuracy of 98.36%. Rohit Kumar Kaliyar and Narang (2021) proposed another model in which BERT embeddings are passed to the CNN model for the classification purpose and after this combination author achieved the accuracy of 98.90%.

### Summary

We observed in the literature review that propagation, linguistic, semantics of text, and user profiles are important metrics for fake news classification. However, we observed two limitations.

1. Researchers used ML and DL methods for the fake news detection considering the local context only and ignoring the global context of text data.
2. The state-of-the-art models used a single dataset and missed a generalized model performance evaluation on heterogeneous datasets.

The MCred model proposed in this paper uses CNN for the local context and BERT for the global context of the given information. Originally, the BERT model has large number of parameters ranging from 100 millions to 300 millions (Devlin et al. 2018). Therefore, the BERT model training from scratch using small dataset leads to over-fitting problem. To avoid this, we used pre-trained BERT model and further trained it on our dataset for fine-tuning. There are three possible ways of fine-tuning: (i) training of complete architecture, (ii) training of some layers of pre-defined architecture, and (iii) use of complete architecture as it stands. We followed the third way in our proposed model and fine-tuned the BERT model by adding our dataset with the pre-existing dataset and also concatenated few additional layers. We explained the implementation details in Sect. 4.2.2. After tuning, we tested their performance using extensive experiments on four heterogeneous datasets.

## Background

### ML background

We used five popular ML methods in developing our proposed MCred model.

*Logistic regression (LR)* evaluates categorical problems. Popular version of LR model have binary result; either true/false, yes/no and other. Instead of this multinomial LR is also available with multiple results. LR takes the advantage of logistic or Sigmoid function to read the input vector and map it to the appropriate category. In this paper we used LR for the evaluation purpose because it robust and flexible method for classification (Seufert 2014).

*Naive Bayes (NB)* classifies the news as real or fake using maximum conditional probability. It is based on “Bayes’ Theorem”.

$$\begin{aligned} P(X|Y) = \frac{P(Y|X) * P(X)}{P(Y)} \end{aligned}$$

where X and Y are two events.

We used the NB classifier because it is simple and computationally inexpensive for text classification. NB needs a lesser amount of data for training purposes, unlike other classifiers.

*Decision tree (DT)* predicts the final class using the recursive partition of all features present in the training dataset. It represents the dataset as a tree, where nodes represent the features, branches represent the decisions and leaves represent the results. We fed the data as input and progressively partition it into small parts until the result finally labels it as real or fake.

*Random forest (RF)* is a amalgamation of multiple trees therefore it is called as forest. It works for both regression and classification types of use cases. RF prevents over-fitting by using ensemble learning and merging multiple DTs to improve the model accuracy. We used this classifier for faster training and learning of our proposed MCred model. As this paper considered binary problem, all the trees in RF votes for a prediction either 0 or 1 and highest votes are considered as final result of RF.

$$\begin{aligned} {\hat{t}} = \frac{1}{N} \sum _{i=1}^{N} {\hat{t}}(a) \end{aligned}$$

Here ${\hat{t}}$ is the tree prediction, “N” represents the total number of trees present in the forest, “i” is the current tree and “a” is the training data.

*Extreme gradient boosting (XGBoost)* utilizes the concept of supervised machine learning algorithm. The idea of Gradient Boosting Machines (GBM) is used in XGBoost. XGBoost is more powerful in terms of performance and deals with data irregularities. We used this classifier because it accurately predicts the target data by combining the output generated by multiple weak learners.

### Global vector (GloVe) (Jeffrey Pennington 2021)

It is an unsupervised learning algorithm for generating vector of a particular word based on global co-occurrence statistics. The word “GloVe” comes from “Global Vector” and the vector representation generated by this algorithm is known as GloVe word embeddings. This embedding extracts the connection among words from statistics and uses the co-occurrence matrix for finding the semantic relationship. Stanford’s GloVe available in four different versions based on its parameters as shown in Table 1.

**Table 1:** GloVe model training details

Corpora name	Number of tokens (billion)	Vocabulary size	Model size
Wikipedia 2014 Gigaword 5	6	400,000	822 MB
Common Crawl	42	1.9 M	1.75 GB
Common Crawl	840	2.2 M	2.03 GB
Twitter	27	1.2 M	1.42 GB

### Deep learning (DL)

#### BERT

Google researchers proposed the BERT (Devlin et al. 2018) model for natural language processing (NLP) applications. They developed a general-purpose pre-trained model using a huge amount of not annotated text on the Internet to overcome the lack of sufficient training data in NLP tasks. These general-purpose models work with any specific task after fine-tuning and bring good accuracy compared to other models trained on small datasets from scratch. One technical development that separates BERT from other ordinary (bidirectional LSTM) models is its simultaneous bidirectional training.

BERT has two types based on the model architecture: i) $BERT_{Base}$ and ii) $BERT_{Large}$. Table 2 shows that the size of both BERT types uses millions of parameters (110M, 340M) for solving various NLP taks. In this paper, we used $BERT_{Base}$ in proposed model because $BERT_{Large}$ is hard to deploy due to its large size and resource constraint.

**Table 2:** BERT model configuration

Model	Layers	Hidden size	Self attention heads	Parameters
BERT Base	12	768	12	110M
BERT Large	24	1024	16	340M

#### CNN for text

CNN became very popular in image processing applications but demonstrated promising results in NLP research applications in recent years too. Kim (2014) showed that CNN gives excellent text classification results after hyperparameter tuning and trained on 100 billion words extracted from Google News using the word2vec vector representation method. Zhang and Wallace (2017) analyzed the performance of a single layer CNN architecture and concluded it is good for sentence classification and as simple as logistic regression and SVM. Zhang et al. (2015) proposed a CNN architecture for text classification operating at the character level and concluded that CNN models can process text as effectively as image data. However, one can use one-dimensional kernels that slide horizontally over the characters, instead of two-dimensional kernels that slide horizontally and vertically over the image pixels.

#### Dense net

A dense net is a fully connected network of neuron layers, where each layer neuron receives the input from the previous layer neurons and passes it to the next layer neurons. The method finally merges the features coming from the previous layer and generates learning features for further processing. The function used in this layer is the same as for linear layers, but the use of the activation function is different:

$$\begin{aligned} O = f(I \cdot w + b), \end{aligned}$$

where *I* shows the input, *O* shows the output value, *w* is the weight, $\cdot$ is the dot multiplication function applied on input and weight, *b* is the bias for model optimization, and *f* is the activation function.

## Methodology

We present in this section the design and methodology underneath the MCred message credibility model for fake news detection.

### MCred model algorithm

**Fig. 1:**
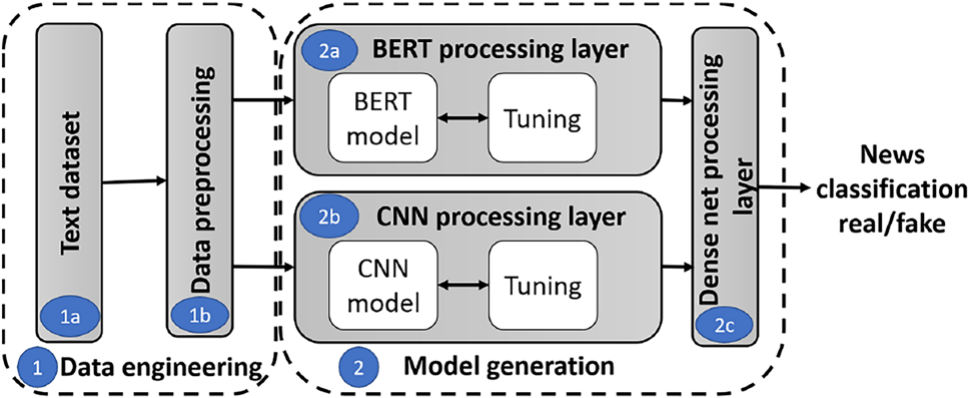
MCred sequence flow diagram

Figure 1 shows the MCred model architecture consisting of two phases implemented in Algorithm 1.

*Data engineering* selects and preprocess a suitable text dataset for the proposed MCred model among several available news datasets in two steps.

1. *Data collection* selects fake news datasets and stores in $MCred\_dataset$ (line 1). A larger dataset prevents the model from over-fitting and enables better model training.
2. *Data preprocessing* performs tasks like noise (e.g. stop word) removal, normalization, and tokenization to keep the data in proper format (line 2).

*Model generation* is a fusion of global and local text semantics for fake news classification consisting of three different sub-layers.

*BERT processing layer*: reads the data from the pre-processing layer and passes it to the BERT pre-trained model tuned for embeddings (line 4). This layer generates global semantics after measuring the relationship among current, previous, and upcoming words in the text. Finally, it passes the output to the dense and dropout layers (line 5).
*CNN processing layer*: reads the pre-processed text data and converts it into GloVe embeddings (line 7). Then, it passes these embeddings through three parallel CNN layers with kernels of sizes two, three, and four (line 8). This layer generates the local text semantics using *n*-gram features and passes the three outputs through multiple dense and dropout layers to produce the final output (line 9).
*Dense net processing layer*: fuses the local and global text semantic outputs from the CNN and BERT processing layers (line 11). It passes the merged outputs to the dense and dropout layers (line 12), and finally produces the news text classification and labels the news as real or fake (line 13).

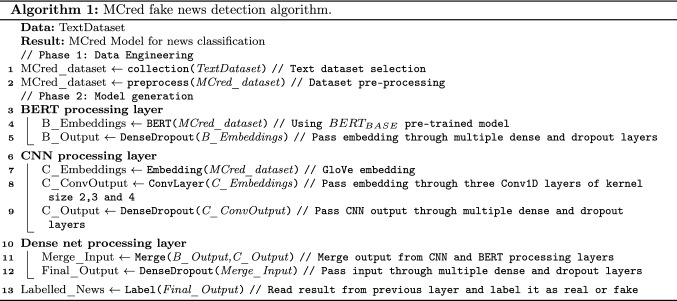



### Proposed MCred model design

#### Data engineering

1. We selected text dataset having equal distribution of real and fake news for better training and evaluation purpose.
2. We pre-processed the raw text dataset through three steps before performing the MCred model training.
  *Tokenization*: breakdowns longer input paragraphs into small sentences. During this process, we protected the sentence delimiters for further execution.
  *Lemmatization*: converts the input words into their canonical form with an equal footing for uniform execution.
  *Stopword removal*: process filtered out stopwords from the input data since its contribution is low as compared to other meaningful data.

#### Model generation

*BERT processing layer:* receives the data from the previous phase and applies three essential data decoration techniques that add metadata to the given text, which is mandatory in the BERT model for the text execution. Initially the training of BERT model is performed on BooksCorpus (Zhu et al. 2015) and English Wikipedia with 800 millions and 2500 millions words respectively. For fine tuning we again train this model with 80% data of our dataset.

**Fig. 2:**
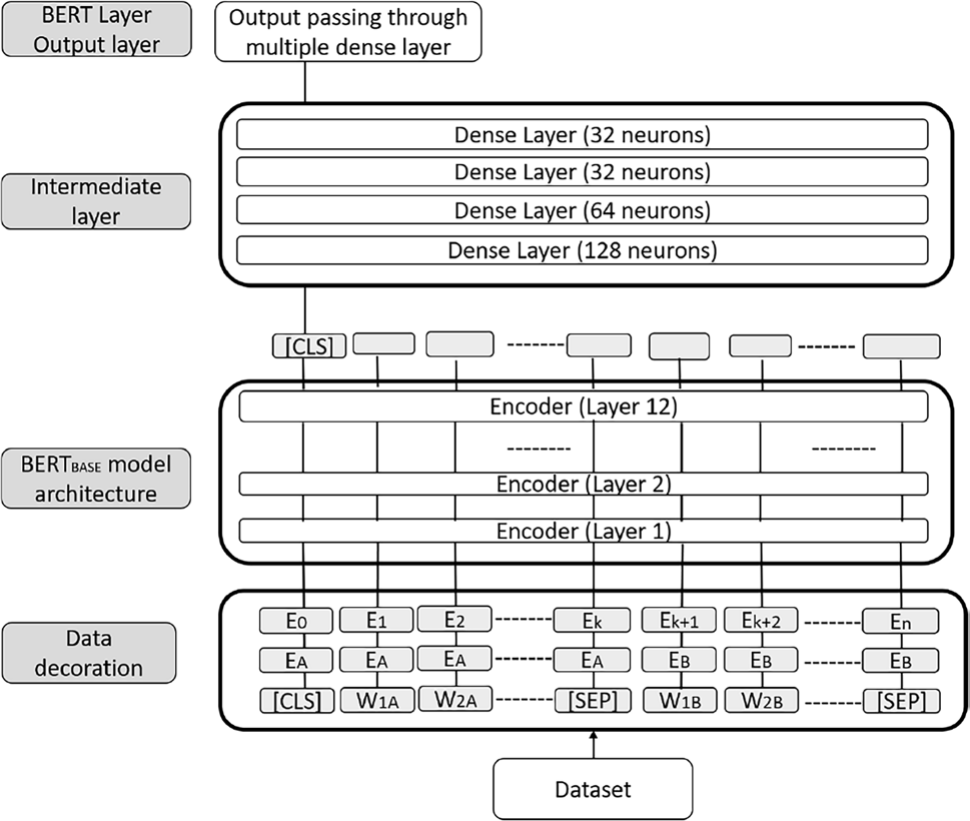
BERT processing layer

*Token embedding*: adds two special tokens, as the data contains multiple sentences: [CLS] token at the beginning of the data and [SEP] token at the end of each sentence. In Fig. 2, $W_{1A}$ and $W_{2A}$ represent the first and second word of the first sentence, while $W_{1B}$ and $W_{2B}$ represent the first and second word of the second sentence.
*Segment embedding*: adds a special marker for different sentences. In Fig. 2, $E_A$ and $E_B$ represent the segment embedding for the first and second sentences.
*Positional embedding*: specifies the token position in the sentence. In Fig. 2, $E_k$ and $E_n$ represent the $k^{\mathrm {th}}$ and $n^{\mathrm {th}}$ elements in the data.

Next, the BERT processing layer converts every token into a 768 long embedding vector, passed further to 12 encoding layers characteristic to the $BERT_{BASE}$ model. The information stored in the [CLS] token is sufficient for classification after processing the twelfth layer. This [CLS] vector flows into the intermediate layer consisting of four dense layers with different neurons. Finally, the BERT processing layer generates the output using a dense layer with 32 neurons.

**Fig. 3:**
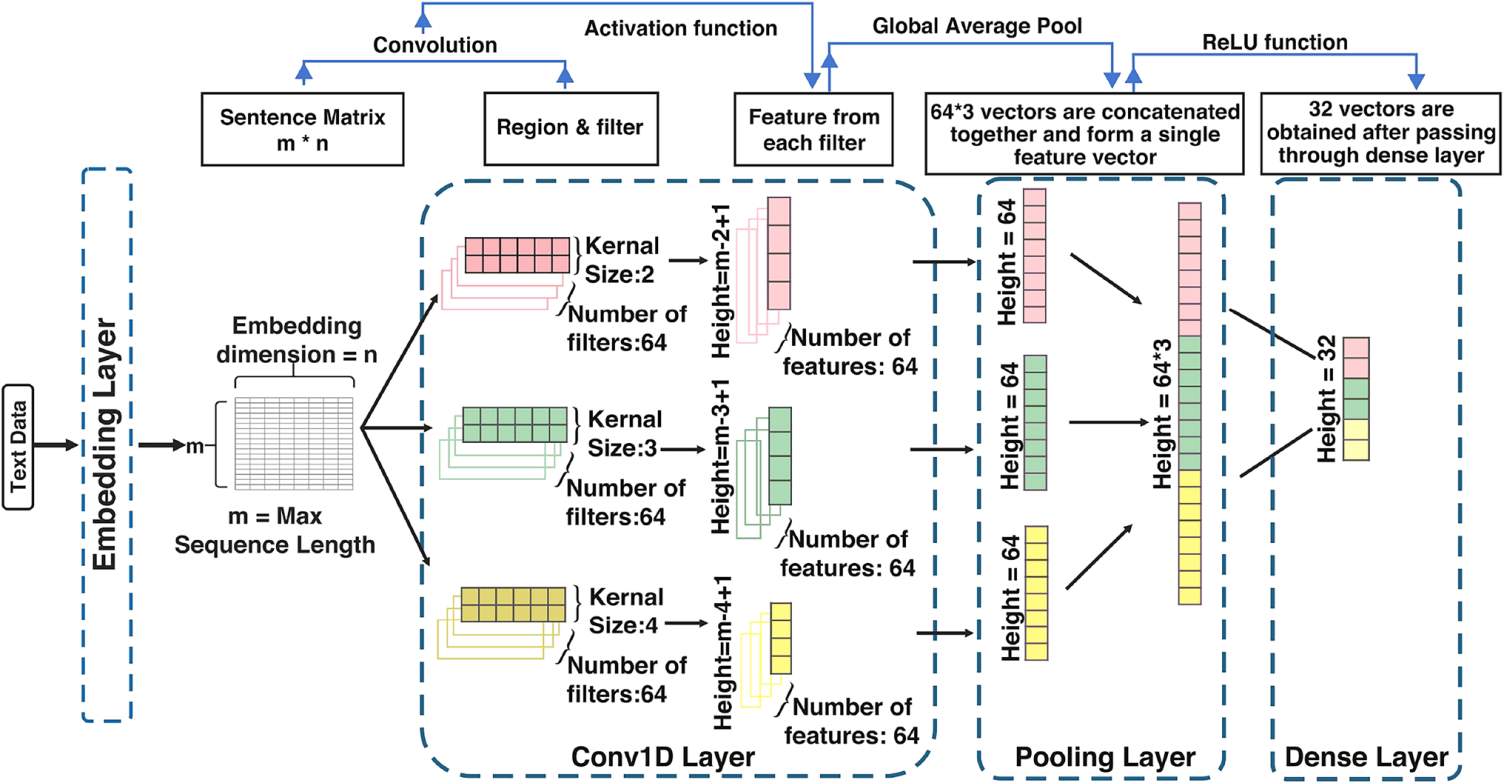
CNN processing layer

*CNN processing layer:* contains four layers as shown in Fig. 3: embedding layer, conv1D layer, pooling layer and dense layer. First, the embedding layer takes and pre-processes the input data and generates the sentence matrix of $m\times n$ size, where *m* is the maximum sequence length and *n* is the embedding dimension. Next, the matrix passes through the one-dimensional convolutional (Conv1D) layer with three 64 filter kernels of sizes two, three, and four. The Conv1D layer generates 64 features from each kernel. The pooling layer processes these three 64 long vectors and concatenates them into a single vector. Finally, the model passes this concatenated vector to the dense layer and converts it into 32 long vectors for next-level processing.

**Fig. 4:**
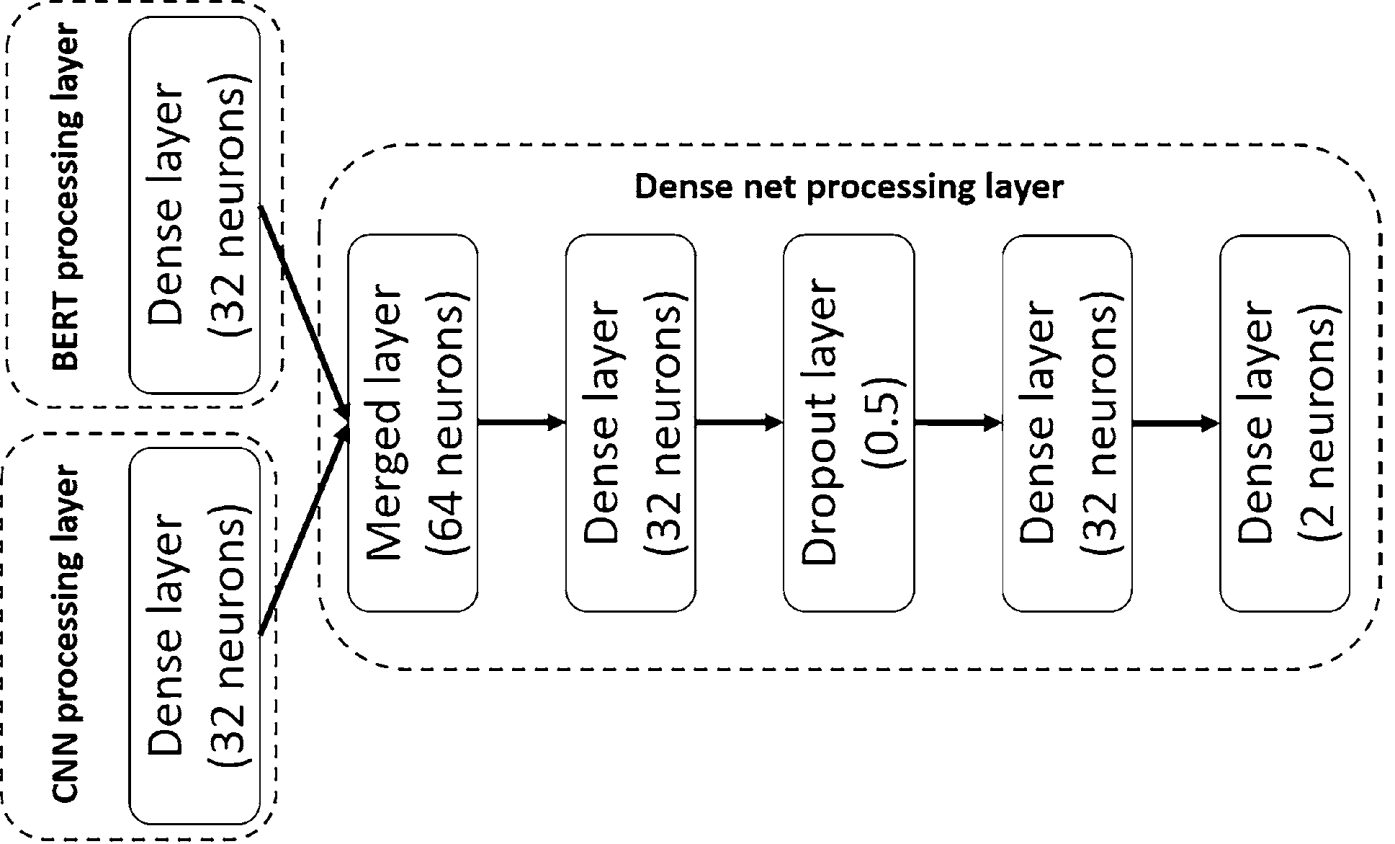
Dense net processing layer

*Dense net processing layer:* combines the 32 long vector outputs of the BERT and CNN layers as shown in Fig. 4, and merges them into a vector of size 64. We used the dropout layer to prevent the over-fitting problem and applied the rectified linear unit (ReLU) activation function at the hidden layers and Sigmoid function at the output layer. After the multiple dense layers, this dense net processing layer generates the final real or fake news classification.

**Table 3:** MCred model architecture

Processing layer	Parameter	Value
*BERT*	Number of dense layers	4
	Dropout rate	0.5
	Activation function	ReLU
*CNN*	Number of dense layers	1
	Number of Conv1D layers	3
	Number of global average pool layers	3
	Activation function	ReLU
	Kernel size	1,2,3
*Dense net*	Number of dense layers	2
	Dropout rate	0.5
	Batch size	64
	Optimizer	Adam
	Activation function	Sigmoid
	Loss	Binary-cross entropy

### Model tuning

We used the random search model tuning technique to examine and improve the MCred model training. We applied the ReLU activation function at both BERT and CNN processing layers. We further used the Adam optimizer and applied a Sigmoid activation function at the dense net processing layer. Table 3 shows the different model tuning parameter details.

*Adam optimizer*(Kingma and Ba 2017) is a memory and computationally efficient enhancement of the gradient descent method that produces improved results in NLP and image processing-based DL applications. This optimizer amalgamates the benefits of AdaGrad and RMSProp optimizers and improves the results with default parameters in various applications.

*ReLU activation function*(Glorot et al. 2011) is simple and offers rapid convergence if sparsely activated. Its better performance over the other activation functions makes it the default option in most network trainings:

$$\begin{aligned} f(x)={\left\{ \begin{array}{ll} 0, &{} x<0.\\ 1, &{} x \ge 0. \end{array}\right. } \end{aligned}$$

*Sigmoid activation function*(Gupta 2020)takes a real value as input and produces an output in the [0,1] interval. This function is non-linear, continuously differential, monotonic, and has a fixed output range:

$$\begin{aligned} f(x) = \frac{1}{1+e^{-x}}. \end{aligned}$$

*Binary cross entropy*(aka *log loss*) (Rajesh and Bhat 2019) deals with the binary problems therefore we used this loss function. The mathematical expression of this function is:

$$\begin{aligned} L= - \frac{1}{N} \sum _{i=1}^{N} \Big ( \big (y_i * log(P(y_i)) \big ) + \big ( (1-y_i) * log(1-P(y_i)) \big ) \Big ) \end{aligned}$$

Where $y_i$ is the actual label and $P(y_i)$ is the probability of data being actual label for all N records.

## Experimental evaluation

We performed several experiments to evaluate the proposed MCred model and compared it with other baseline approaches using a number of relevant metrics described in this section.

### Experimental setup

We implemented the proposed MCred model using sklearn, matplotlib, nltk, and other libraries from the Python 3.9 distribution. We trained our model on workstation with Intel Xeon^®^Gold 5222 3.8GHz processor, 128GB 8*16GB DDR4 2933 RAM, 1TB 7200 RPM SATA hard disk and Windows 10 Pro operating system. We trained our model on both Graphics Processing Unit (GPU) and Central Processing Unit (CPU) and estimated the time required for training process. Table 4 shows the required time in seconds on both processing units.

**Table 4:** Training time

Processing unit	Time (in seconds)
CPU	10,800
GPU	3,600

### Experimental text dataset

We used the four datasets summarized in Table 5 to implement and evaluate the proposed MCred model. We focus our validation on the WELFake dataset (Verma et al. 2021) that reduces the biases and limitations of the others. The WELFake dataset consists of evenly distributed news text data *labeled* as unreliable (1) and reliable (0). The dataset contains fields: news *identifier*, news *title*, and news *text* comprising its *heading* and *content*. Initially, the news text and title fields contained a few undefined values. Therefore, we combined them and created a new information parameter to reduce the undefined values and increase the number of input tokens for improved model training.

**Table 5:** Dataset description

Dataset	Fake news	Real news	Characteristics
Kaggle (Lifferth 2020)	10369	10349	Contains news title, text and author name
McIntire (Hamel ,^2020^)	3164	3171	News articles related to 2016 US presidential election
FakeNews (Risdal 2020)	24396	13614	News collected from heterogeneous sources and topics
WELFake (Verma et al. 2021)	37106	35028	Minimizes the limitations of other individual dataset

### Evaluation metrics

We define four parameters based on the relation between the predicted and the actual news classification, displayed in Table 6: true-positive (TP), true-negative (TN), false-positive (FP), and false-negative (FN). We evaluated the MCred model on four performance metrics based on these parameters.

**Table 6:** Fake news prediction parameters

Evaluation parameter	Predictive value	Actual value
True-positive (TP)	Yes	Yes
True-negative (TN)	No	No
False-positive (FP)	Yes	No
False-negative (FN)	No	Yes

*Accuracy* is the ratio between the number of correct predictions and the total number of predictions:

$$\begin{aligned} Accuracy = \frac{TP+TN}{TP+TN+FP+FN}. \end{aligned}$$

*Precision* measures the positive predicted value, as the ratio between the number of correct positive predictions to the total number of positive predictions:

$$\begin{aligned} Precision = \frac{TP}{TP+FP}. \end{aligned}$$

*Recall* *R* measures the sensitivity of the as the ratio between the number of correct positive predictions to the total number of correctly predicted results:

$$\begin{aligned} Recall = \frac{TP}{TP+FN}. \end{aligned}$$

*F1-score* measures the testing accuracy of the model as the harmonic mean of the precision and the recall:

$$\begin{aligned} F1\textit{-}score = \frac{2}{ Recall ^{-1} + Precision ^{-1}}. \end{aligned}$$

### Experimental results

In this section, we analyze the results achieved by the MCred model using the tuning process presented in Sect. 4.3 and the parameters in Table 3. We performed our experiments on an 80 : 10 : 10 train-test-validation split.

**Table 7:** MCred model results on various optimizers

Parameter	Optimizer	Val_Loss	Val_Acc	Testing dataset			
				Accuracy	Precision	Recall	F1-Score
Dropout (0.5)	Adam	**0**.**0160**	**0**.**9959**	**0**.**9901**	**0**.**9921**	**0**.**9882**	**0**.**9901**
	SGD	0.3898	0.8299	0.8258	0.8220	0.8161	0.8190
	RMSProp	6.9984	0.5107	0.5071	0.5071	0.9901	0.6729
	Adagrad	0.5159	0.7620	0.7587	0.7530	0.7477	0.7503
Dropout (0.3)	Adam	0.0442	0.9858	0.9852	0.9823	0.9881	0.9851
	SGD	0.3905	0.8208	0.8164	0.8718	0.7199	0.7886
	RMSProp	0.1408	0.9553	0.9481	0.9410	0.9516	0.9463
	Adagrad	0.4551	0.7854	0.7844	0.7831	0.7680	0.7755

#### Dropout and optimizer selection

Table 7 compares the results using two dropout values (0.3 and 0.5) on four optimizers: Adam, SGD, RMSProp, and Adagrad. Interestingly, the increase in dropout consistently improved the MCred model performance on all evaluation parameters with the Adam and SGD optimizers, while it compromised the performance with RMSprop and Adagrad. The Adam optimizer outperforms the others due to its combination of RMSProp and Adagrad optimizers to handle sparse gradients on large and noisy data. The Adam optimizer produces better results due to its fewer memory requirements and small learning rate adapted to individual parameters for sparse datasets. The higher dropout improves the overall performance of the MCred model by reducing the validation loss to 1.60% and maximizing the validation accuracy to $99.59\%$, the testing accuracy to $99.01\%$, the precision to $99.21\%$, the recall to $98.82\%$, and the F1 score to $99.01\%$.

#### Learning curve

**Fig. 5:**
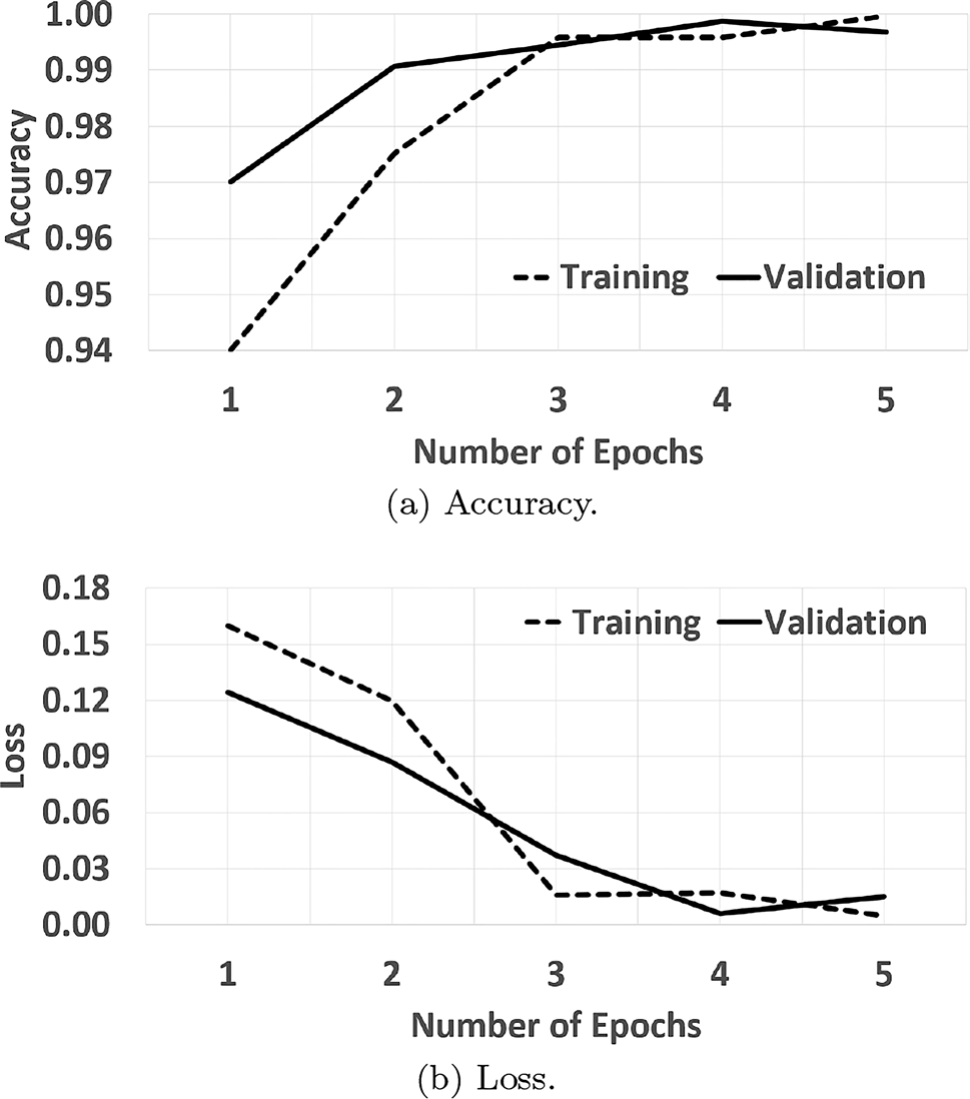
Model accuracy and loss.

We further analyzed the MCred model performance by drawing a learning curve between the training and validation data. Figure 5 shows two learning curves at different epochs: accuracy and loss. Initially, the gap between validation and training data in both curves was very high. After the execution of five epochs, the model reduced this gap and became stable, demonstrating good fit condition (i.e. always between overfitting and underfitting) for two reasons. The gap between training and validation loss is minimum at the stable point.

#### MCred versus ML models

We compared proposed MCred model with both ML and DL models as sometimes ML models also perform better. We implemented LR, NB, DT, RF, and XGBoost models on the WELFake dataset and tuned them on several hyper-parameters as shown in Table 8. Then, we compared their performance with the proposed MCred model. We extracted text features using the GloVe word embedding technique and converted the text into a feature vector. We fed this vector into these models and analyzed their performance in Table 9. The accuracy of various ML models ranged between $89.46\%$ and $97.65\%$. Among the five ML models, XGBoost outperformed with $97.65\%$ accuracy followed by RF, DT, NB, and LR. Although, XGBoost achieved a remarkable performance in terms of accuracy yet it was 1.36% lower than the proposed MCred model. It clearly shows that the fusion of several deep learning methods used in the proposed MCred model improved the accuracy as compared to other ML models.

**Table 8:** Hyper-parameters for ML models tuning

Model	Parameter	Value
LR	C Penalty Solver	0.01 l2 lbfgs
NB	Smoothing Fit prior	1 true
DT	Maximum features Criterion Cost complexity pruning	auto gini 0.02
RF	n_estimators	50
XGBoost	n_estimators learning_rate	100 0.01

**Table 9:** MCred model performance comparison with other ML models

Model Name	Accuracy (%)
Logistic Regression (LR)	89.46
Naive Bayes (NB)	92.38
Decision Tree (DT)	93.56
Random Forest (RF)	94.12
XGBoost	97.65
**MCred**	**99.01**

#### Comparison of MCred with other DL models

Our proposed model is based on BERT-CNN architecture but for the performance evaluation we compared the performance of our model with other deep learning fusions too. For this, we implemented two separate models BERT-RNN and BERT-LSTM on same dataset i.e., WELFake dataset. RNN (Olah 2021) is different from traditional neural networks because it uses the the output obtained from previous step as the input for the next step and it remembers the past information too. Among various types of RNN i.e., one-to-one, one-to-many, many-to-one and many-to-many, we used many-to-one because it is suitable for classification task. LSTM (Olah 2021) is a type of RNN that designed to overcome the limitation of RNN like; (i) gradient vanishing and exploding, (ii) complex training and (iii) difficulty to process very long sequences. Table 10 clearly shows that for the text classification problem BERT-CNN model is excessively suitable and as per the architecture of RNN and LSTM both are suitable for other tasks like question-answering, machine translation etc.

**Table 10:** MCred model performance comparison with other DL models

*Model Name*	*Accuracy (%)*
BERT-CNN	99.01
BERT-RNN	94.56
BERT-LSTM	96.94

**Table 11:** Comparison of MCred with state-of-the-art methods

	Mersinias et al. (2020)	Khan and Alhazmi (2020)	Kaliyar et al. (2020)	Rohit Kumar Kaliyar and Narang (2021)	MCred
Dataset accuracy	Kaggle: 97.52% McIntire: 94.53% FakeNews: 96.78%	Kaggle: 90.70%	Kaggle: 98.36%	Kaggle: 98.90%	Kaggle: 99.46% McIntire: 97.16% FakeNews: 97.98% WELFake: 99.01%
Document representation features	Class label frequency distance vector	Doc2Vec	GloVe	BERT embeddings	GloVe – BERT embeddings
Classifier	Logistic regression (ML) CNN + LSTM (DL)	AdaBoost LinearSVM	Deep CNN	CNN	CNN, BERT

### State-of-the-art comparison

Table 11 compares MCred with three recent state-of-the-art works; Mersinias et al. (2020), Khan and Alhazmi (2020), Kaliyar et al. (2020) and Rohit Kumar Kaliyar and Narang (2021).

- Mersinias et al. (2020) used a content-based approach for fake news detection. They combined logistic regression with a deep learning model and achieved $97.52\%$ accuracy on the Kaggle dataset.
- Khan and Alhazmi (2020) used Doc2Vec features for fake news classification and applied two ensemble learning techniques: Bagging-LinearSVM and AdaBoost-LinearSVM. They achieved a maximum accuracy of up to 90.7%.
- Kaliyar et al. (2020) proposed the FNDNet model based on GloVe word embedding using a deep CNN method on Kaggle dataset. They achieved the highest accuracy up to 98.36%.
- Rohit Kumar Kaliyar and Narang (2021) proposed FakeBERT model that reads BERT embeddings as input and gives the improved accuracy of 98.90%.

Amalgamation of local and global text semantics and use of BERT pretrained model in MCred model makes it different and efficient among other models. In Mersinias et al. (2020), Khan and Alhazmi (2020) and Kaliyar et al. (2020) only local semantics are used but on the other hand Rohit Kumar Kaliyar and Narang (2021) used BERT embedding and passed to the CNN for the classification. For a fair comparison, we trained and tested the MCred model on the same three datasets used by other researchers: Kaggle, McIntire, and FakeNews. We used $80\%$ of each dataset for training, $10\%$ for testing, and $10\%$ for validation. Table 11 clearly shows that the MCred model improves the accuracy not only with the WELFake dataset but also achieves better accuracy of 99.46%, 97.16%, and 97.98% on Kaggle, McIntire, and FakeNews datasets respectively.

### MCred model summary

We used WELFake dataset to classify the real and fake news using message credibility. For this, we proposed an MCred model which is a fusion of two DL methods (*i.e.*, CNN and BERT). Then we implemented five ML models on the same dataset and compared the performance. Further, we implemented two fusions of DL models (*i.e.*, BERT-RNN and BERT-LSTM) and compared their performance with the proposed MCred model. We also compared our proposed model with other recent state-of-the-art works and found that the proposed model outperformed over the other state-of-the-art works. The proposed model gives better accuracy but it has few following limitations: *i)*The complexity of self-attention layer at training is $O(n^2)$, ‘n’ is the sequence length,ak points during training and testing phase. Therefore BERT processing model takes more time for large inputs. *ii)* CNN processing layer requires large data to train and it is slower because of maxpool operation. Similarly at the testing phase, we require properly preprocessed and larger data.

## Conclusions and future work

We proposed a new model called MCred model to classify the text news as real or fake using the global and local semantic relationship among the words. We modeled the local semantic relationships using a CNN with kernel sizes of two, three, and four, and the global semantic relationships using a pre-trained BERT model. The new MCred model combines the outputs of BERT and CNN models and processes them in a dense network layer for final prediction. Experimental results revealed an MCred accuracy of 99.46% on the Kaggle dataset, 97.16% on the McIntire dataset, 97.98% on the fake news dataset, and 99.01% on the WELFake dataset. With respect to state-of-the-art research, MCred achieved an accuracy improvement of 1.94% compared to Mersinias et al. (2020), 8.76% compared to Khan and Alhazmi (2020), and 1.10% compared to Kaliyar et al. (2020) on the Kaggle dataset. MCred further achieved better accuracy than Mersinias et al. (2020) by 2.63% on the McIntire dataset and by 1.2% on the FakeNews dataset.

We plan to extend our work in the future with more features based on user credibility, knowledge graphs, and propagation analysis. Image-based news analysis and Deepfake analysis are also in our attention.

## Data Availability

We implemented proposed MCred model on WELFake dataset in the manuscript and it is available at: https://doi.org/10.5281/zenodo.4561253. We used other open source dataset for analysis purpose and cited them in the manuscript.
